# Semi-supervised contour-driven broad learning system for autonomous segmentation of concealed prohibited baggage items

**DOI:** 10.1186/s42492-024-00182-7

**Published:** 2024-12-24

**Authors:** Divya Velayudhan, Abdelfatah Ahmed, Taimur Hassan, Muhammad Owais, Neha Gour, Mohammed Bennamoun, Ernesto Damiani, Naoufel Werghi

**Affiliations:** 1 Department of Electrical Engineering and Computer Sciences, Center for Cyber-Physical Systems, Khalifa University of Science and Technology, Abu Dhabi, 127788 United Arab Emirates; 2 Department of Electrical, Computer and Biomedical Engineering, Abu Dhabi University, Abu Dhabi, 59911 United Arab Emirates; 3 Department of Computer Science and Software Engineering, the University of Western Australia, Perth, WA 6009 Australia

**Keywords:** Baggage X-ray imagery, Broad learning systems, Threat detection, Threat segmentation

## Abstract

With the exponential rise in global air traffic, ensuring swift passenger processing while countering potential security threats has become a paramount concern for aviation security. Although X-ray baggage monitoring is now standard, manual screening has several limitations, including the propensity for errors, and raises concerns about passenger privacy. To address these drawbacks, researchers have leveraged recent advances in deep learning to design threat-segmentation frameworks. However, these models require extensive training data and labour-intensive dense pixel-wise annotations and are finetuned separately for each dataset to account for inter-dataset discrepancies. Hence, this study proposes a semi-supervised contour-driven broad learning system (BLS) for X-ray baggage security threat instance segmentation referred to as C-BLX. The research methodology involved enhancing representation learning and achieving faster training capability to tackle severe occlusion and class imbalance using a single training routine with limited baggage scans. The proposed framework was trained with minimal supervision using resource-efficient image-level labels to localize illegal items in multi-vendor baggage scans. More specifically, the framework generated candidate region segments from the input X-ray scans based on local intensity transition cues, effectively identifying concealed prohibited items without entire baggage scans. The multi-convolutional BLS exploits the rich complementary features extracted from these region segments to predict object categories, including threat and benign classes. The contours corresponding to the region segments predicted as threats were then utilized to yield the segmentation results. The proposed C-BLX system was thoroughly evaluated on three highly imbalanced public datasets and surpassed other competitive approaches in baggage-threat segmentation, yielding 90.04%, 78.92%, and 59.44% in terms of mIoU on GDXray, SIXray, and Compass-XP, respectively. Furthermore, the limitations of the proposed system in extracting precise region segments in intricate noisy settings and potential strategies for overcoming them through post-processing techniques were explored (source code will be available at https://github.com/Divs1159/CNN_BLS.)

## Introduction

Recently, X-ray baggage screening has become the standard in security surveillance, strengthening aviation security measures to cope with the evolving risks posed by sophisticated threats brought about by the continuous surge in global air traffic, while ensuring swift passenger processing [1]. However, the current reliance on manual assessment presents inherent limitations, including the propensity for errors owing to tediousness and fatigue [2]. The need for quick identification of diverse prohibited items, including firearms and liquids beyond pre-set limits, adds to the complexity, particularly during peak hours. Furthermore, with the additional challenges of occlusion, overlapping contours, and scale variations, existing screening procedures require both continual vigilance and extensive expertise [3]. Studies [4] also indicate that human operators can achieve only 80%−90% success, which is critical considering the consequences. To overcome these shortcomings, authorities worldwide have encouraged developing robust automated baggage and cargo-screening systems [5, 6]. Early research focused on traditional image processing, and machine learning approaches suffered from suboptimal performance owing to heavy reliance on hand-engineered features [7–9]. By contrast, with recent advancements in computer vision empowered by deep-learning algorithms, researchers have shifted their focus toward state-of-the art object detection and segmentation models [10, 11] to develop autonomous baggage threat recognition frameworks.

However, the performance of these models has been unsatisfactory owing to the inherent disparity between X-ray and RGB imagery, which is attributed to their texturelessness, limited pseudo-colors, and contrast, which is further worsened by the skewed distribution of contraband items and severe occlusion by high-density objects in baggage [8, 12]. Moreover, current frameworks are influenced by variations in imaging hardware and scanner properties [13]. Recent research addresses only particular challenges, focusing on edge and contour cues for occlusion [14–16], hierarchical features, and attention mechanisms [17], as well as data sampling [18] and algorithmic level techniques [19] for class imbalance, and generative models [20]. Despite these advancements, these frameworks rely on large, densely annotated datasets that require extensive skilled labor [21, 22]. Additionally, few frameworks addressing occlusion have been tested on a single dataset [17, 23] or are highly dataset-dependent [14, 24]. Approaches that use 3D object detection with volumetric baggage computed tomography (CT) imagery [25] are inefficient and rely on limited training datasets. These methods are fully supervised, necessitating well-annotated data that are challenging to obtain, particularly for rare suspicious items.

Furthermore, most current frameworks for X-ray baggage threat detection and segmentation rely on deep convolutional neural networks (CNNs), which require extensive training and significant computational resources. These models have complex structures with millions of parameters tuned via backpropagation and are often dataset-dependent, necessitating separate training for each dataset, resulting in long training times and frequent remodeling [26–28]. Alternatively, the broad learning system (BLS), introduced by Chen and Liu [29], maps input to a feature space using arbitrary weights, allowing faster training [30]. BLS has a universal approximation capability and performs well in tasks such as facial emotion recognition [31], hyperspectral image classification [32], and human activity recognition [33].

However, the shallow modeling of BLS limits its feature learning capability. Integrating BLS with high-level semantics from whole-bagging X-ray scans using deep neural nets may be ineffective because of irrelevant overlapping background objects and severe occlusion. X-ray images are a superimposition of threatened items and benign background content, where the contours of different objects are blended. Unlike photographic images, X-ray scans contain pixels that belong to multiple categories, which complicates feature extraction [34]. Using entire scans as inputs to deep neural networks extracts irrelevant background features along with threat features, leading to distraction. Studies have shown that segmenting baggage scans into coarse segments yields better threat-identification results than using entire scans [35, 36].

Hence, this study proposes a semi-supervised contour-driven road learning system for X-ray baggage security threat instance segmentation, referred to as C-BLX. This system offers enhanced representation learning and faster training capability to effectively identify and localize illegal items in multi-vendor baggage scans, addressing severe occlusion, overlap, and class imbalance using a single training routine. The proposed framework was trained with minimal supervision using resource-efficient image-level labels rather than the labour-intensive dense pixel-level annotations utilized by other competing threat segmentation models [24, 27]. The recursive candidate refinement (RCR) block integrated into the framework addresses occlusion and improves the identification of concealed and barely visible threats. It generates candidate region segments from input X-ray imagery by exploiting intensity variations using multi-directional tensors. Additionally, the substantial number of region segments generated by the framework enables training on a balanced training set, effectively mitigating class imbalance concerns and minimizing the baggage scans required for training. Furthermore, unlike other competitive approaches in baggage-threat segmentation, which need to be finetuned separately on different datasets, the proposed framework can effectively identify concealed and cluttered contrabands using a single training instance from limited baggage scans, irrespective of the variations in the scanners. The contributions can be summarized as follows:

- *Minimal supervision requirement:* The proposed framework is trained with minimal supervision using resource-efficient image-level labels rather than dense pixel-level annotations, unlike competing supervised threat segmentation models that heavily rely on large, labor-intensive mask-level training annotations.
- *Segment-based approach:* The proposed C-BLX framework utilizes a segment-based method of segmenting the entire baggage scan into candidate regions, which helps better identify concealed threats, as opposed to using entire scans, which often include irrelevant background noise. Experimental results (impact of region segment extraction on performance subsection) further validate these capabilities. The capacity of the model to extract meaningful segments from the input enables it to focus on these regions rather than processing the entire image, thereby conserving computational resources.
- *Computational efficiency:* In contrast to state-of-the-art (SOTA) baggage threat segmentation frameworks that are finetuned separately on different datasets, the proposed C-BLX system utilizes a single training instance to identify concealed contraband from baggage scans regardless of the variations in the scanning properties.
- *Performance improvements:* The ability of RCR to generate candidate region segments from the input X-ray imagery enables the framework to identify prohibited items effectively from the region proposals rather than whole baggage scans, also allowing the framework to be less susceptible to the intrinsically skewed distribution of abnormal scans (comprising of baggage threat items). This advantage is further highlighted by the comparative analysis in Comparative results subsection, which demonstrates the superiority of our methods over other competitive approaches that disregard the skewed distribution. Our model achieves higher accuracy, mean Dice coefficient (*µ*DC), and mean intersection-over-union (*µ*IoU) (detailed in Evaluation metrics subsection) compared to other SOTA models.

### Related works

#### BLS

The BLS, originally based on the compressed sensing technique and pseudo-inverse theorem [30], has attracted considerable attention because of its proven universal approximation capability and faster computational ability [32, 37]. Unlike functional link neural networks [38], which use iterative gradient descent, BLS sets weights randomly based on a probabilistic distribution, thus reducing computational time. It transforms inputs into an appropriate feature plane via random mappings that are fine-tuned using sparse regularization [37] to enhance data handling. Unlike deep-learning models, BLS maintains a shallow architecture with enhancement nodes, enabling accurate task execution without extensive training on large-scale data. Structurally, BLS comprises input, features, and enhancement nodes connected through weighted links. The input data are transformed into random features and projected using a nonlinear activation function to form enhancement nodes [39]. The output layer weights were obtained through a pseudo-inverse estimation [40]. Various BLS variants have been proposed using autoencoders for sparse weights, wavelets for feature mapping, fuzzy systems to replace mapped features, and random hierarchical features to enhance representation learning [29, 30, 41, 42].

#### X-ray baggage threat segmentation

##### Supervised threat segmentation strategies

Early methods in X-ray baggage threat segmentation relied on transfer learning and fine-tuning SOTA object segmentation models [36, 43], while later works incorporated attention mechanisms within encoder-decoder frameworks to enhance performance [34, 44]. Some frameworks explored edge cues with trainable structure tensors to localize subtle and concealed prohibited items [14, 15]. For instance, Hassan et al. [15] used an encoder-decoder model that leveraged multiscale contour features, whereas Wei et al. [23] integrated edge and material information with object detectors to identify occluded threats. Shafay et al. [45] explored boundary cues to capture relevant semantic features while suppressing extraneous information. Ma et al. [34] employed a dense attention-based instance segmentation framework guided by high-level semantic features.

Few frameworks have focused on robust feature learning for occluded contrabands using multiscale feature aggregation and refinement techniques. Shafay et al. [27] used multi-level features in a lightweight framework and improved them using temporal cues from consecutive scans [45]. Chouai et al. [46] proposed adversarial autoencoders for semantic segmentation, and Hassan et al. [3] developed an incremental learning encoder-decoder model. Wang et al. [21] enhanced the detection performance using adaptive dense attention modules [47], and Sara and Mandava [48] leveraged spatial and channel attention for threat segmentation. Recently, Liao et al. [49] explored a threat recognition approach that integrated feature enhancement using frequency-domain knowledge. Meanwhile, refs. [28, 50] used the contextual features from transformers to localize threats. Nasim et al. [51] proposed a multi-instance threat-segmentation framework to detect overlapping threat instances without additional overhead. However, these studies did not consider inherent class imbalance issues in X-ray baggage security screening, where benign objects significantly outnumber threat instances.

To address the class imbalance, Miao et al. [17] proposed a custom loss with hierarchical features, and Ahmed et al. [19] used an instance segmentation framework with a novel loss function. Researchers have also explored volumetric CT images for 3D segmentation and detection; however, practical deployment remains challenging owing to the low efficiency, high fine-tuning, and scalability overheads, and cost of CT imagery [25]. Furthermore, most current frameworks rely heavily on large amounts of densely annotated data and lack comprehensive validation across multiple datasets, which is a notable limitation.

##### Semi-supervised threat segmentation strategies

A few studies [52, 53] leveraged adversarial networks to detect anomalous scans (those containing threats). Some studies [35, 54] attempted to decompose the entire scan into coarse subcomponents to identify anomalous objects within semantically benign baggage items. Bhowmik et al. [35] utilized iterative clustering methods to generate superpixel segments from input scans, which were then classified as abnormal or benign. They also found that their results outperformed those of object-level segmentation.

##### Unsupervised threat segmentation strategies

In ref. [54], deep learning-based unsupervised clustering within a joint segmentation classification pipeline was used to identify anomalous items. Hassan et al. [55] employed an encoder-decoder framework trained only with benign scans for unsupervised threat segmentation, using discrepancies between the original input and reconstructed imagery and Fourier stylization to suppress scanner dependencies.

Existing approaches cannot handle imbalanced real-world data [43, 44] or strong dataset dependency, with limited validation across multiple datasets [27, 46]. Most semi-supervised and unsupervised frameworks are tested on proprietary datasets, limiting transparency and generalizability and not addressing challenges owing to scanner variability. A notable gap is the lack of frameworks capable of one-time training with minimal samples to recognize concealed contrabands in X-ray images, regardless of the scanner specifications [54]. C-BLX can train faster with limited resources, thus addressing the identified limitations. Recent work on baggage threat detection has studied the endogenous shift in X-ray security screening owing to intrinsic factors, such as imaging mechanisms and hardware components [5]. They used a lateral inhibition mechanism to focus on relevant features and capture boundary and shape information. Our approach aligns with this concept by concentrating on structural features within tightly encapsulated region segments, enabling the model to better discern item shapes. This targeted approach allows our model to generalize well for a few scans across different datasets.

## Methods

**Fig. 1:**
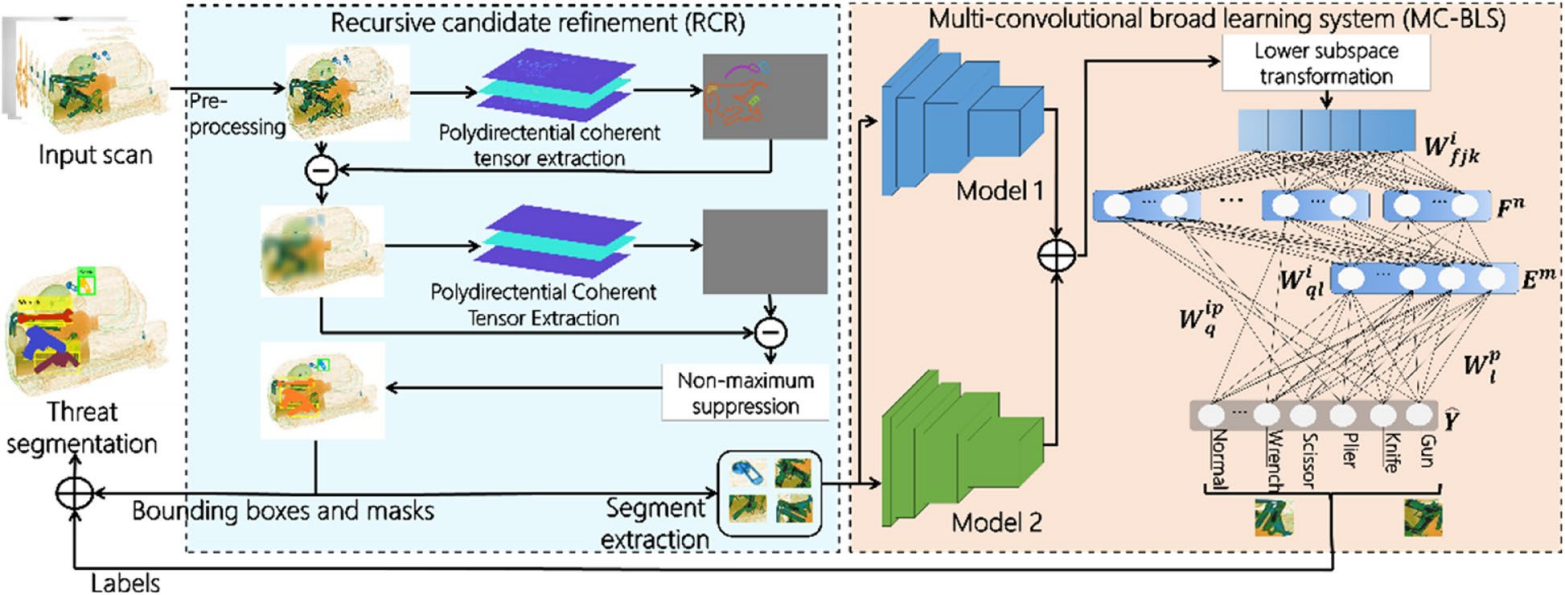
Block diagram of the proposed C-BLX framework. The RCR block employs the polydirectional coherent tensor extraction approach recursively to extract the candidate segments containing items of varying density effectively from the input scan. These candidate region segments are passed to the CNN backbones, trained via cross-entropy loss, to extract features that are concatenated and eventually classified by the BLS. Detected threats are localized using bounding boxes of the region segments identified as prohibited items, and masks are created from the contours generated by RCR corresponding to these threat objects, ensuring precise segmentation

This section provides an overview and detailed explanation of the proposed C-BLX system, a semi-supervised contour-driven BLS designed for the segmentation of security threats in X-ray baggage scans, as illustrated in Fig. 1. The C-BLX framework comprises two main modules: the RCR block and the multi-convolutional broad learning system (MC-BLS), along with the implemented training strategy.

**Fig. 2:**
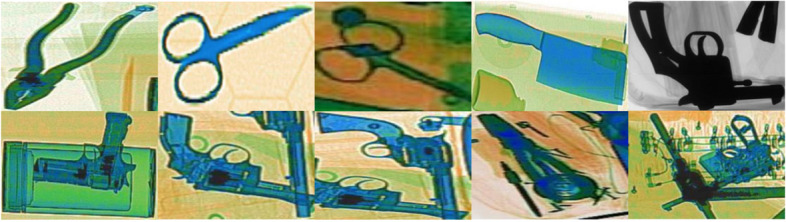
Top row shows extracted region segments with isolated objects, while the bottom row shows segmented regions containing occluded, merged and cluttered objects

The RCR block serves as the initial processing step, utilizing advanced image processing techniques to highlight contours and local patterns within the X-ray scans to produce discrete regional segments, efficiently isolating areas of potential interest that may contain prohibited or benign objects (Fig. 2 for examples of extracted candidate region segments). These candidate region segments are passed to an MC-BLS with multiple varied CNN backbones to extract pertinent and diverse features, substantially boosting the representation learning ability of the proposed framework. These extracted features are fused and subjected to principal component analysis (PCA) to transform them into a lower-dimensional subspace, reducing the computational burden and eliminating redundant features, while maximizing the distinction between feature distributions across various categories. The BLS architecture uses these transformed features to predict the class labels of the objects contained within the extracted region segments. After filtering out the candidate segments containing the background and other benign baggage items, the contour maps produced by the RCR for the threat identified segments were used for segmentation. In the testing phase, the RCR block generates regional segments from new scans, which are then processed by a trained CNN backbone to extract features. These features were classified using BLS to predict the object categories within the test scans. The objects identified as threats were localized using the bounding boxes of their respective region segments, with contour maps facilitating accurate segmentation, as illustrated in Fig. 1.

The term ‘semi-supervised’ is loosely used to indicate that only limited supervision is employed via image-level class labels, avoiding reliance on dense pixel-level annotations commonly required by conventional methods. This distinctive choice of supervision aims to streamline the annotation process and resource requirements. Our training strategy involves deliberately using a considerably small subset of the training dataset, in accordance with established protocols, followed by other competitive methods. Specifically, scans from the training subsets of three distinct datasets (SIXray, GDXray, and COMPASS-XP) were consolidated, their standard protocols were adhered to, and a limited subset of these scans was utilized for subsequent training. The proposed C-BLX system can recognize different categories of suspicious and benign items across multiple datasets using a single training routine constrained by the categorical cross-entropy (L_*CE*_) with minimal supervision in the form of cost-effective class labels. Generating several candidate segments from a single input scan guarantees sufficient and balanced training data, making the framework immune to class imbalance issues inherent in the domain, while limiting the number of scans used in the training routine.

### RCR

The RCR serves as a critical component within the framework and employs a recursive approach to remove the candidate segments by exploiting the contours and local intensity transition cues (refer to Fig. 2 for examples of extracted segments). The region segments generated from each X-ray scan encompassed both normal and prohibited baggage items. The recursive methodology makes the framework better at identifying concealed threats of varying densities, from high-density metallic threats, such as handguns, to barely visible ones, such as razor blades. Generating multiple candidate segments from each scan also ensures a balanced training set, effectively addressing class-imbalance issues. RCR also supports training with limited data, which is beneficial given the challenge of obtaining sufficient training data for X-ray baggage threat identification because the task is sensitive.

The initial step involves preprocessing the input scans for contrast enhancement using adaptive histogram equalization [56] to better reveal the item contours. The modified scans were subjected to a polydirectional coherent tensor extraction approach (Algorithm 1) to highlight the contours using modified structure tensors [18] by analyzing local patterns based on* L* gradients associated with* L* different orientations, where *L* ∈ *N*. This yields an *L* ×* L* polydirectionally structured tensor block, as shown in Eq. 1.

1 $$\rho = \left[\begin{array}{ccc}{\rho }_{0}^{0}& {\rho }_{1}^{0}& {\rho }_{L-1}^{0}\\ {\rho }_{\begin{array}{c}0\\ .\\ .\\ .\end{array}}^{1}& {\rho }_{\begin{array}{c}1\\ .\\ .\\ .\end{array}}^{1}& {\rho }_{\begin{array}{c}L-1\\ .\\ .\\ .\end{array}}^{1}\\ {\rho }_{0}^{L-1}& {\rho }_{0}^{L-1}& {\rho }_{L-1}^{L-1}\end{array}\right]$$

Each element *ρ*^*l*^_*k*_ is a tensor representation encapsulating the local structure at a point based on its neighborhood, obtained from the outer product of the gradient vectors oriented along the *k*^*th*^ and *l*^*th*^ directions and weighted by the smoothing function.

**Fig. 3:**
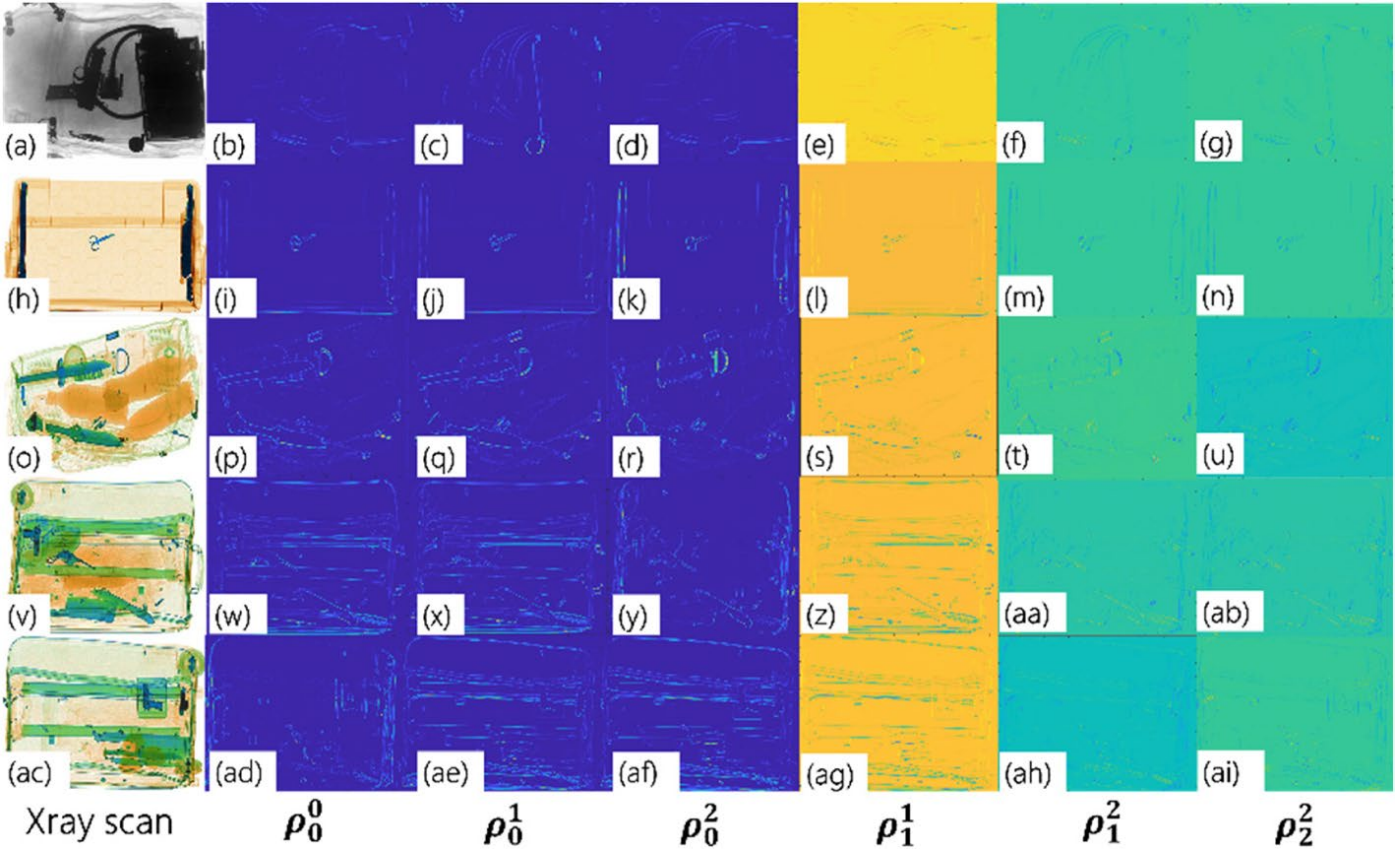
Six unique tensor representations yielded for *L* = 3. Each row corresponds to a different scan, with the first column showing the original scan and the subsequent columns displaying tensor maps. These maps, arranged from left to right, represent $\rho_0^0,\;\rho_0^1,\;\rho_0^2,\;\rho_1^1,\;\rho_1^2,\;\rho_2^2$, obtained by computing image gradients along the angles 0, $\frac{2\pi}3$ , and $\frac{4\pi}3$

Thus, *ρ* is symmetric (because $\rho\frac lk=\rho\frac kl$), yielding $\frac{{\mathcal{L}}\left({\mathcal{L}}+1\right)}2$ unique representations. The gradients are computed along the direction $\vartheta=\frac{2\pi r}{\mathcal{L}}$ where *r* varies from 0 to *L* − 1. For example, *L* = 3 yields six unique tensor representations from three image gradients oriented at 0, $\frac{2\pi}3$ rad, and $\frac{4\pi}3$ (Fig. 3 using randomly chosen baggage scans). The tensor maps in the first column ($\rho_0^0$ ) are obtained from the image gradients computed along 0 rad, whereas those in the second column ($\rho_0^1$ ) are derived from the image gradients computed along 0 rad and $\frac{2\pi}3$ rad. Similarly, those in the third column $\left(p_0^2\right)$ were obtained from the gradients computed along 0 and $\frac{4\pi}3$ radians.

Furthermore, coherent tensor selection was performed to reduce computational overhead and noise by selecting T prime tensors with maximal coherency based on their norm. These tensors were merged into a single coherent tensor ˜*ρ*. Singular value decomposition was used to decompose non-square tensors into components, facilitating the selection of the most coherent tensor with the maximum eigenvalue strength. The optimal values of *L* and *T* are determined empirically in the ablation analysis (refer to Ablation study subsection). The coherent tensor ˜*ρ* is then binarized and subjected to connected-component analysis [57]. A bounding rectangle was constructed for each labeled component, tightly encapsulating the labeled region with minimal width and height. After non-maximum suppression, these bounding boxes were used to extract the corresponding region segments from the input X-ray scan.

However, applying the polydirectional coherent tensor extraction approach only once is ineffective for X-ray scans, as materials with varying densities exhibit different intensity gradients that are further dependent on the overlapping materials. High-density objects (metallic weapons) have more pronounced edges than low-density objects (e.g., razor blades), leading to potential extraction failures. Thus, the extraction approach was applied recursively to account for varying intensity gradients. In the RCR block, the previously extracted segments are discarded, and the corresponding pixels are interpolated using Dirichlet boundary values. This process is repeated until all labeled components are extracted. The generated regional segments may contain single or multiple items, particularly in cluttered scans. Segments with a single suspicious item or part of it are labeled by that class, whereas segments without suspicious items were labeled as Normal. Segments with overlapping suspicious items were labeled by the class spanning the largest area. These candidate region segments, along with their segment-level labels, were used to train the MC-BLS, which extracted relevant occlusion-resistant high-level features and enhanced the ability of the framework to identify concealed and subtle baggage threats.

### MC-BLS

The MC-BLS, comprised of *N* CNN backbones and BLS, predicts the respective object categories utilizing the deep, low-rank features from the extracted candidate region segments of the input baggage scans. CNNs were employed as the backbone to extract rich, high-level semantics and diverse complementary features from each segment. These features are more pertinent because they are obtained from regional segments that encapsulate different baggage items, unlike the features extracted from whole baggage scans, which can be confused with overlapping contours. CNN backbones are crucial for overcoming the limitations of the BLS’s shallow structure, expanding its ability to identify various suspicious items. By integrating features from parallel connected CNNs, the framework captures diverse aspects of input data, resulting in richer and more robust feature representations. This ensemble approach leverages the diversity and complementarity of individual CNN backbones, potentially enhancing the overall performance and robustness of threat detection. In the proposed framework, the value of *N* is empirically set to 2. This choice is supported by two justifications: (1) the significant increase in the number of computational parameters as *N* exceeds two, and (2) the achievement of satisfactory and competitive results for *N* ≤ 2 (as demonstrated in Ablation study subsection), whereas the performance improvement was not substantial for *N* > 2.

Suppose *B* = {*B*_1_*, B*_2_} represent the lightweight CNN feature extractors, trained using the dataset D = {*X,Y*}, where *X* ∈ R^*M*×*J*^ represents the input (with the number of samples *M* and channels *J*), and *Y* ∈ R^*M*×*C*^ represents the class labels (with *C* number of classes), to extract distinct latent features *f* = ^P2^_*i*=1_ *f*_*i*_, where *f*_*i*_(*X*) = Φ(*B*_*i*_(*X*)) is obtained from the *i*^*th*^ feature extractor, Φ(*.*) denotes the mapping function, and *B*_*i*_(*X*) = *w*_*i*_*X* + *β*_*i*_, with weights *w*_*i*_ and bias *β*_*i*_.

The latent features *f*_*i*_(*X*) extracted from the respective backbones are concatenated to form a rich and diversified set of representations (*f*) capable of distinguishing between different suspicious item categories. However, concatenating features increase the dimensionality, leading to issues such as poor generalization, extreme overfitting owing to the curse of dimensionality [58], and high computational costs. To address this issue, *f* is decomposed into a low-rank orthonormal matrix using a subspace decomposition block. The subspace decomposition block maps higher-dimensional data to lower-dimensional data using PCA, thereby reducing the number of redundant computations. The latent representations are mapped: *L* ∈ R^*M*×*G*^, where *M* denotes the input data samples, and *G* represents the cumulative feature representations from the *B* backbones to a subspace S ∈ R^*M*×*V*^, where *V* ≪ *G*. Each data sample *x* ∈ R^1×*l*^ in *L* is projected to S as ˆ*x* ∈ R^1×*V*^, with ˆ*x* = *u*^*T*^*e* + *µ*, where $\mu=\;\frac1M\;{\textstyle\sum_{h=0}}^{-1}\;x_h$^*M*^ is the mean of *x*, *u* = {*u*_1_*,u*_2_*,…,u*_*V*_} are the orthonormal eigenvectors, and *e* = {*e*_1_*,e*_2_*,…,e*_*V*_} are the eigenvalues such that *e* = *u*^*T*^(*x* − *µ*). A subset of eigenvectors ˆ*u* = {*u*_1_*,u*_2_*,…,u*_*v*_} is used, where 0 < *v* < *V*, to project *L* into a low-rank subspace, significantly reducing the latent space dimension.

This transformation is crucial for removing redundant and irrelevant data while retaining distinct and relevant features, thereby maximizing the variance between the feature distributions of different categories. Furthermore, it allows the BLS to reap the benefits of rich feature representations to perform underlying classification tasks while significantly reducing the features’ dimensional space. The transformed feature representations are then passed on to the BLS to identify different suspicious items.

**BLS:** The BLS, an integral part of the proposed instance segmentation framework, utilizes the deep, low-rank, distinct, and relevant features extracted from the region segments to predict the respective object categories. Let *X* denotes the input nodes that are connected with *n* mapped features blocks *F*^*N*^ = {*F*^1^*,F*^2^*,F*^3^*,…,F*^*n*^}, with *k* nodes in each block via weight matrix *W*_*F*_ and a biasing factor *β*_*F*_, as expressed below:

2 $$\begin{array}{cc}F^i\:=\:\phi_i\left({XW}_{Fi}\:+\:\beta_{Fi}\right),&\;i\:=\:1,2,3,\dots,n\end{array}$$

where *W*_*Fi*_ and *β*_*Fi*_ are randomly set and *ϕ*_*i*_ is the mapping function (the most chosen ones are linear and kernel mappings) for the *i*^*th*^ group of mapped features *F*^*i*^. The feature nodes are connected to the *m* enhancement nodes *E*^*M*^ = {*E*^1^*,E*^2^*,E*^3^*,….,E*^*m*^} computed as follows:

3 $$\begin{array}{cc}E^j\:=\:{\mathfrak I}_j(F^nW_{Ej}\:+\:\beta_{Ej}),&j\:=\:1,2,3,\dots,m\end{array}$$

where *W*_*Ej*_ and *β*_*Ej*_ denote the weight matrix and biasing factor, respectively; and ℑ_*j*_ represents the mapping function, which may include convolution, nonlinear activation, or kernel mapping. The feature and enhancement nodes are then connected to the output nodes *Y*^ˆ^ via *W*^*m*^-weighted links. The output *Y*^ˆ^ is computed as follows:

4 $$\hat Y=\;\lbrack\phi1({XW}_{F1}\;+\;\beta_{F1}),...,\phi n({XW}_{Fn}\;+\;\beta_{Fn})\vert{\mathfrak I}_1(F^nW_{E1}\;+\;\beta_{E1}),...,\mathfrak Im(FnWEm\;+\;\beta Em)\rbrack Wm$$

5 $$\begin{array}{ll}\hat Y\;&\begin{array}{c}=\;\lbrack F^1,...,F^n\vert{\mathfrak I}_1(F^nW_{E1}\;+\;\beta_{E1}),...,{\mathfrak I}_m(F^nW_{Em}\;+\;\beta_{Em})\rbrack W^m\end{array}\\&=\;\lbrack F^1,...,F^n\vert E^1,...,E^m\rbrack W^m\\&=\;\lbrack F^N\vert E^M\rbrack W^m\\&=\;AW^m\end{array}$$

where *W*^*m*^ = A^+^*Y* and *W*^*m*^ represents the weights of the BLS architecture used for computing its output *Y*ˆ. These weights are computed using the ridge regression approximation of A^+^ as follows:

6 $${}_{W^m}^{min}:\;\left\|{AW}^m\;-\;\hat Y\right\|_2^2\;+\;\lambda\;\left\|W^m\right\|_2^2$$

*λ* is a regularization parameter that puts further constraints on to sum of the squared weights *W*^*m*^. Moreover, equating the derivative of Eq. 6 to zero yields:

7 $$W^m\;=\;\frac{A^T\hat Y}{\left(\lambda I\;+\;{AA}^T\right)}$$

where the weights of the BLS system (*W*^*m*^) can be tuned from Eq. 7 because the matrix (*λI* + AA^*T*^) is non-singular [32]. Specifically, $A^+\;=\;{}_{\lambda\rightarrow0}^{lim}\left(\lambda I\;+\;{AA}^T\right)^{-1}\;A^T$, where *I* is the identity matrix.

### Instance threat segmentation

**Fig. 4:**
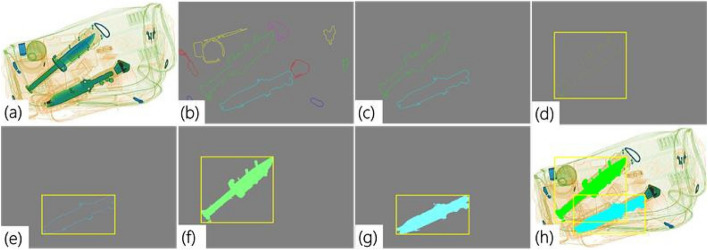
Visualization of threat detection process: **a** Original scan; **b** Generated coherent tensor map highlighting object boundaries and local patterns; **c** Objects post non-maximum suppression; **d**, **e** Regions of interest after rectangle fitting; **f**, **g** Mask generation by filling inner pixels; and **h** Final threat segmentation overlaid on the original image

The proposed framework leverages low-rank diverse representations extracted from rectangle-bounded regions localized by RCR, such as the segmentation of prohibited items from cluttered, overlapping baggage imagery. The candidate segments predicted by the BLS as background and other benign baggage items were filtered out, whereas the spatial coordinates of the rectangular bounding boxes of the region segments containing the prohibited items were used to localize the detected threat objects. Furthermore, the contours generated by RCR corresponding to the threat objects within these segments were used to generate masks. If the contours are not closed or intact, the endpoints of the open contours are joined together and the inner pixels are filled to generate the corresponding masks (Fig. 4).

#### Single-instance training

The C-BLX framework employs a single training procedure with weak supervision using cost-effective class labels to recognize various prohibited items across multiple datasets, regardless of scanner variations. Unlike SOTA models that require separate fine-tuning of different datasets with detailed annotations, C-BLX uses single-instance training, significantly reducing the training and evaluation times. This approach demonstrates the ability of the model to detect concealed contrabands across diverse datasets without requiring dataset-specific fine-tuning.

To ensure robustness and generalizability, three datasets were used: SIXray, GDXray, and COMPASS-XP. The SIXray dataset includes scans from three different types of scanners: GDXray provides grayscale images of baggage scans and COMPASSXP offers diverse representations of the same baggage scan, including low-energy and high-energy X-ray images, density images, color images, grayscale images, and RGB images. The framework was trained with considerably fewer samples from each dataset to address the challenge of acquiring ample training data for X-ray baggage threat identification. This is achieved by generating several candidate segments from a single input scan to ensure sufficient and balanced training data. Initially, the pretrained CNN models were fine-tuned to identify various contraband categories. During training, these models use a categorical cross-entropy loss to learn distinct feature representations. The training was performed once, and the heads were removed to extract high-level semantic representations for subsequent stages. The BLS model weights are tuned via random approximations from the sparse autoencoder, with weights converging quickly, eliminating the need for expensive backpropagation.

## Results and Discussion

### Datasets

The proposed framework has been evaluated on three publicly available datasets: GDXray [59], SIXray [17], and COMPASS-XP, using only a small fraction of their standard training subsets. The following is a summary of these datasets:

**Figure:**
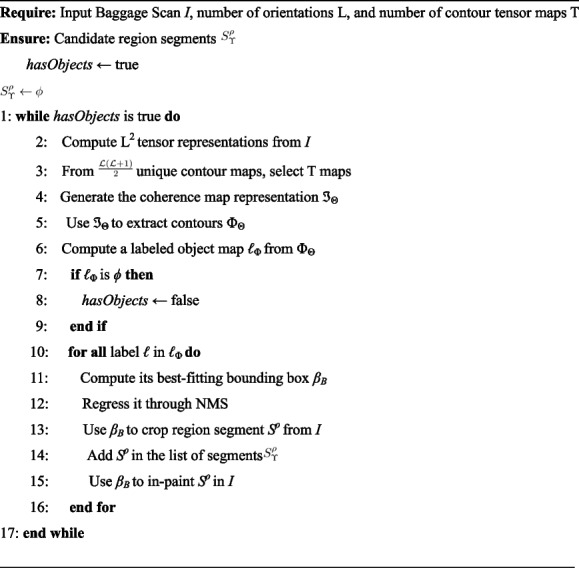
**Algorithm 1** Proposed RCR approach

*GDXray* [59] contains 8,150 images of baggage scans from a total of 19,407 grayscale X-ray images. This includes bounding box annotations for *shuriken*, *knives*, *guns*, and *razors*.

*SIXray* [17] is the largest pseudo-colored baggage X-ray dataset with 1,059,231 scans, including 8,929 positive scans annotated with bounding boxes for items such as *knives*, *wrenches*, *guns*, *pliers*, *hammers*, and *scissors*. It addresses class imbalance through the subsets SIXray10, SIXray100, and SIXray1000. Each subset adhered to the dataset standards, with 80% of the scans allocated for training. However, only a few training scans are used to finetune the framework, as detailed in Implementation details subsection.

*Compass-XP* [60] includes 11,568 scans with diverse representations such as low energy and high-energy X-ray, density, color, grayscale, and RGB images. It has 1928 images per representation with 258 threat images, making it valuable for evaluating binary classification and abnormality identification frameworks.

### Implementation details

The training phase of our framework was conducted in MATLAB R2022b on a computer equipped with an Intel(R) Core(TM) i7-10750H CPU operating at a base frequency of 2.60 GHz. The system also boasts 24 GB of RAM and a single NVIDIA GTX 1660Ti GPU. The training of the CNN backbones was performed over 50 epochs, deploying a batch size of 128. The Adam [61] optimizer was used, with an initial learning rate of 0.001, complemented by a momentum of 0.9. The regularization parameter *λ* for ridge regression was set to 10^–3^, whereas the weight matrices *W*_*F*_ and *β*_*F*_ of the feature node block were drawn from a standard uniform distribution over the interval [-1*,*1]. The weights of the BLS were computed using ridge regression, as shown in Eq. 7. In total, an average of 167 segments was extracted from each training scan, taken from a set of 5,800 scans across all three datasets, with each segment resized to 224 × 224 pixels. Highly cluttered scans may result in more segments, whereas less cluttered scans may yield fewer segments. Adhering to the respective data-splitting protocols across all datasets, 80% of the scans were allocated for training and 20% for testing. In addition, 10% of the training subset was set aside as the validation set for hyperparameter tuning and model refinement. We selected 5,500 scans from the standard training subset of the SIXray dataset, 200 from the GDXray dataset, and 100 from COMPASS-XP. Owing to this rigorous training and data preparation process, 860,000 object-region segments were generated. To address the potential class imbalance issues in the proposed C-BLX model, a balanced stream was carefully curated. This stream comprised 60,000 segments representing seven suspicious items and 8,126 segments representing normal baggage content, ensuring a comprehensive representation of diverse scan types.

### Evaluation metrics

We utilized several metrics to assess our model’s performance in recognizing suspicious items: Mean average precision (*µ*AP), *µ*IoU, *µ*DC, and accuracy. In this study, true positives (*T*_*p*_) represents correctly identified contraband pixels, false positives (*F*_*p*_) represents background pixels misclassified as positive, and false negatives (*F*_*n*_) represent contraband pixels misclassified as background pixels.

IoU quantifies the pixel-level overlap between model predictions and the ground truth, calculated as:

8 $$IoU\;=\;\frac{T_p}{T_p\;+\;F_p\;+\;F_n}$$

The *µ*IoU is obtained by averaging IoU scores for each contraband class.

The dice coefficient (DC) assesses performance in identifying contraband items, calculated as follows:

9 $$DC\;=\;\frac{{2T}_p}{{2T}_p\;+\;F_P\;+\;F_n}$$

The *µ*DC was obtained by averaging the DC scores of each item category.

*µ*AP is calculated by taking the mean of the average precision (AP) score for each threat class, with an IoU threshold ≥ 0.5:

10 $$\mu AP\;=\;\frac1{nc}\;\sum\limits_{i=0}^{nc-1}\;AP(i)$$

where *nc* denotes the number of contraband item classes.

Classification accuracy quantifies the proportion of correct predictions out of the total input samples, represented as follows:

11 $$Accuracy=\;\frac{T_p\;+\;T_n}{T_p\;+\;F_n\;+\;T_n\;+\;F_p}$$

### Ablation study

The ablation study for the proposed framework covers several key components: (1) selection of orientation for tensor computation and coherent tensor selection, based on the hyperparameters *L* and *T*; (2) choice of the optimal ensemble backbone models for feature representation; (3) impact of various activation functions in the BLS, which identified SeLU as the top performer; (4) identifying the suitable BLS parameters; (5) analyzing the impact of region segment extraction over entire baggage scans on the overall model performance, demonstrating significant enhancement in threat detection capabilities and computational efficiency.

#### Number of gradient directions and tensor selection

We evaluated the framework by varying the number of gradient directions *L* from two to four and the number of selected tensor maps *T* from 1 to L*(L + 1)/2 (these hyperparameters are relevant to the RCR block, as detailed in RCR subsection) and calculated the AP. As Table 1 displays, there is a general improvement trend as* L* increases. Notably, for* L* = 3, peak performance was observed at *T* = 2, with AP scores of 95.2% and 97.1% for the GDXray and SIXray datasets, respectively, indicating that this setting offers the best balance between orientation and tensor selection. The performance declines for *L* > 3, which can be attributed to the inclusion of additional tensors that introduce spikes and erratic transitions, thereby generating noisy edges and undesired regional segments.

**Table 1:** Detection performance in terms of AP by varying the number of orientations (*L*) and selected tensor maps (*T*) for GDXray and SIXray

*L*	**Tensor maps **(*T*)	**GDXray**	**SIXray**
2	1 2 3	0.601 0.683 0.773	0.617 0.761 0.825
**3**	1 2 3 4 5 6	0.667 **0.952** 0.651 0.824 0.742 0.781	0.746 **0.971** 0.692 0.868 0.759 0.843
4	1 2 3 4 5 6 7 8 9 10	0.801 0.942 0.930 0.893 0.756 0.621 0.547 0.378 0.309 0.253	0.829 0.968 0.902 0.892 0.704 0.585 0.516 0.409 0.231 0.182

#### Optimal ensemble feature extractors

To identify the most efficient and accurate backbone model, various models including ResNet-50 [62], GoogleNet [63], MobileNetv2 [64], VGG-16 [65], EfficientNet-B0 [66], DenseNet201 [67], and Xception [68] were evaluated. Each model is assessed for accuracy and parameter count, as shown in Table 2. Although DenseNet201 and Xception show high accuracy, they require extensive computational resources. EfficientNet-B0 achieved the highest accuracy with 3.71 times fewer parameters than DenseNet201. Thus, EfficientNet-B0 was chosen for its balance between performance and computational efficiency as an ensemble pair in our C-BLX framework.

**Table 2:** Choice of the backbone models

Model	Accuracy	Parameter
ResNet-50 [62]	0.9462	33.2 M
GoogleNet [63]	0.9532	6.80 M
MobileNetv2 [64]	0.9255	**4.62 M**
VGG-16 [65]	0.9469	86.3 M
**EfficientNet-B0 ** [66]	**0.9766**	5.39 M
DenseNet201 [67]	0.9684	20.0 M
Xception [68]	0.9679	22.8 M

EfficientNet-B0 was combined with various models to determine the optimal ensemble pair. As shown in Table 3, EfficientNet-B0 and ResNet-50 achieved the highest accuracies of 99.46%. Despite a larger parameter count (38.59 M), the computational cost remained reasonable. Other combinations, such as EfficientNet-B0 with Xception or DenseNet201, did not satisfactorily balance the accuracy and computational cost. Although EfficientNet-B0 with MobileNetv2 showed a manageable performance, its accuracy was lower. Thus, the EfficientNet-B0 and ResNet-50 combination was selected as the optimal backbone model for further experimentation.

**Table 3:** Comparison of EfficientNet-B0 with different models

Backbone1	Backbone2	Accuracy	Parameter
EfficientNet-B0	VGG-16	0.9711	91.69 M
**EfficientNet-B0**	**ResNet-50**	**0.9946**	38.59 M
EfficientNet-B0	GoogleNet	0.9707	12.19 M
EfficientNet-B0	MobileNetv2	0.9688	**10.01 M**
EfficientNet-B0	DenseNet201	0.9834	25.39 M
EfficientNet-B0	Xception	0.9805	28.19 M

#### Optimal activation functions in BLS

We analyzed the impact of different activation functions on BLS performance, evaluating ReLU, SeLU, PReLU, Tansig, Sin, Softsign, Tanh, and Sigmoid. As shown in Table 4, SeLU achieved the highest accuracy of 93.32%, surpassing that of Tansig by 0.61%. Therefore, SeLU was selected for the BLS because of its superior performance.

**Table 4:** Performance comparison of different activation functions

Activation function	Accuracy
ReLU	0.9253
**SeLU**	**0.9332**
PReLU	0.9251
Tansig	0.9275
Sin	0.9226
Softsign	0.9250
Tanh	0.9253
Sigmoid	0.6907

#### BLS parameter tuning

**Fig. 5:**
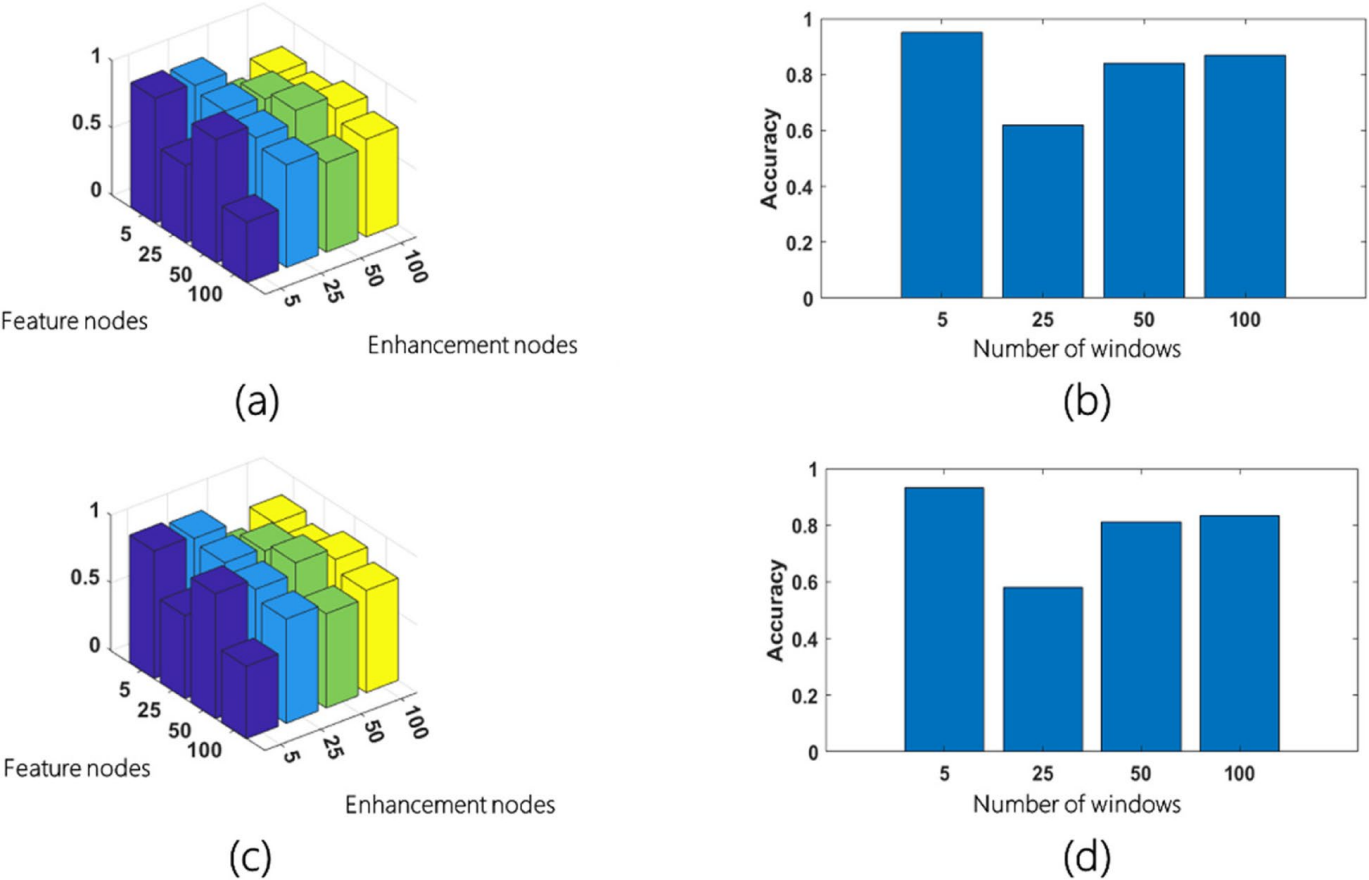
BLS Parameter Optimization. **a** and **c** represent the performance variation with different numbers of enhancement nodes and feature nodes per window using SIXray and GDXray, respectively; **b** and **d** show the accuracy achieved with different numbers of windows of feature nodes for the SIXray and GDXray datasets, respectively

We also performed ablation studies using SIXray and GDXray datasets to examine the interplay between three BLS parameters: number of enhancement nodes, feature nodes per window, and number of windows of feature nodes. As shown in Fig. 5a and c, performance peaks on both GDXray and SIXray datasets, respectively, when the number of enhancement nodes and feature nodes per window is 5, yielding accuracies of 94.31% and 92.81%, respectively. With the optimal number of enhancement nodes and feature nodes per window identified, the last parameter—the number of windows of feature nodes—was fine-tuned. In this study, as shown in Fig. 5b for the SIXray dataset and Fig. 5d for the GDXray dataset, shows that the highest accuracies are achieved when the number of windows is 5 for both datasets, leading to accuracies of 93.06% and 95.17%, respectively. The optimal parameter configuration balances the complexity and performance.

#### Impact of region segment extraction on performance

As shown in Table 5, extracting region segments from whole baggage scans significantly improves threat detection performance. This approach increased the accuracy from 93.09% to 94.98% for GDXray, from approximately 3.08% for SIXray, and from 59.73% to 61.21% for COMPASS-XP. These results confirm that region segmentation enhances the ability of the model to effectively identify threats by focusing on meaningful segments rather than processing the entire image and saving computational resources.

**Table 5:** Performance comparison with and without region segment

Approach	GDXray	SIXray	COMPASS-XP
**Using region segments (proposed)**	**0.9498**	**0.8567**	**0.6121**
Using the whole scan	0.9309	0.8311	0.5973

We also compared our proposed method with alternative methods for extracting region segments, such as region proposal networks (RPNs). RPNs, however, RPNs must be trained explicitly on X-ray security screening datasets and require ground-truth bounding box information. This also involves hyperparameter tuning to ensure optimal learning, which results in excessive computational time. As shown in Table 6, our approach is 28 times faster than faster RCNN with fewer parameters and multiply-accumulate operations (MACs). MACs are a measure of computational complexity, representing the number of arithmetic operations required, and directly impact model size and computational resources. This rapid computational capability and lower resource requirements render our framework an efficient solution for real-world scenarios, allowing easy adaptability to emerging threats without laborious annotations.

**Table 6:** Comparative analysis of training time

Approach	Training time (s)	Parameter (M)	MACs (G)
Faster RCNN [10]	19,359	41.5	134.6
Proposed	680	30.8	4.5

## Comparative results

We conducted a comprehensive evaluation of our proposed threat segmentation framework against SOTA studies in X-ray baggage screening using multiple datasets. SOTA baggage-threat segmentation frameworks are finetuned separately on different datasets, whereas the proposed C-BLX system utilizes a single training instance to identify concealed contrabands from baggage scans regardless of the variations in the scanning properties. The results summarized in Table 7 demonstrate the considerable improvements achieved by our framework. Specifically, on the SIXray dataset, our model outperformed the second-best scheme, CIE-Net, by improving *µ*DC by 3.01% and *µ*IoU by 14.7%. On the GDXray dataset, the proposed model surpassed the leading model by 5.77% in terms of *µ*DC, and by an impressive 16.6% in terms of *µ*IoU. The percentage improvementis calculated using the formula:

12 $$\mathrm{Percentage}\;\mathrm{improvement}\;=\left(\frac{\mathrm{Performance}\;\mathrm{metric}\;(\mathrm{our}\;\mathrm{model})-\;\mathrm{Performance}\;\mathrm{metric}\;(\mathrm{CIE-}\mathrm{Net})}{\mathrm{Performance}\;\mathrm{metric}\;(\mathrm{CIE-}\mathrm{Net})}\;\ast\;100\right)$$

**Table 7:** Performance comparison between our proposed model and other SOTA threat detection frameworks

**Method**	**Dataset**	*µ***IoU**	*µ***DC**	*µ***AP**
**Proposed**	GDXray	**0.9004**	**0.9218**	**0.9613**
	SIXray	**0.7892**	**0.8399**	**0.8221**
	COMPASS-XP	0.5944	0.7636	-
	Combined	0.4995	0.7022	-
CIE-Net [3]	GDXray	0.7723	0.8715	0.8556
	SIXray	0.6883	0.8153	0.7653
	Combined	**0.5861**	**0.7390**	**0.7249**
MS RCNN [69]	GDXray	0.7201	0.8372	0.8091
	SIXray	0.6484	0.7867	0.6756
	Combined	0.5482	0.7081	0.6298
Mask RCNN [70]	GDXray	0.7098	0.8302	0.7833
	SIXray	0.6381	0.7790	0.6326
	Combined	0.5243	0.6879	0.5983
HTC [71]	GDXray	0.7364	0.8481	0.8481
	SIXray	0.6559	0.7921	0.7384
	Combined	0.5804	0.7344	0.7203
YOLACT [72]	GDXray	0.7089	0.8296	0.7478
	SIXray	0.6110	0.7585	0.6237
	Combined	0.4937	0.6610	0.5937
DSRL [73]	GDXray	0.7421	0.8519	-
	SIXray	0.6542	0.7909	-
	Combined	0.5709	0.7268	-
MvRF-CNN [74]	GDXray	0.6982	0.8222	-
	SIXray	0.6016	0.7512	-
	Combined	0.4918	0.6593	-
TST [14]	GDXray	0.6851	0.8131	-
	SIXray	0.5874	0.7400	-
	COMPASS-XP	0.8202	0.9014	-
	Combined	0.4285	0.5999	-

Furthermore, our proposed model employed a combination of SIXray, GDXray, and COMPASS-XP datasets, while all other SOTA models used a combination of only SIXray and GDXray datasets, as shown in Table 7. Despite these differences in the dataset composition, C-BLX surpassed several other SOTA models. This achievement underscores the robustness and adaptability of the proposed framework under disparate conditions. Our framework trails the leading CIE-Net by approximately 14.8% in terms of *µ*IoU. However, it is crucial to consider the significant differences in the training conditions between the proposed approach and CIE-Net. CIE-Net benefits from a more extensive training dataset (27,750 scans from only two datasets: GDXray and SIXray) and employs dense pixel annotations (masks) in a fully supervised setting. In contrast, our model was trained on a more constrained dataset of 5,800 scans from three datasets (GDXray, SIXray, and COMPASS-XP) using only image-level class labels for training.

### Comparison of run-time performance and model complexity

Our proposed model demonstrates superior threat segmentation performance and excels in runtime efficiency. As shown in Table 8, our model was the fastest among the compared methods. Although models such as YOLOv3 prioritize speed, they occasionally miss accurate threat extraction, which is crucial. Although models such as Mask R-CNN, MS R-CNN, and HTC offer high accuracy, they are significantly slower. Our framework balances accuracy and speed and provides efficient threat item extraction. To further illustrate the efficiency of the proposed model, the model complexity was compared in terms of MACs and the number of parameters. Table 8 shows that our approach requires fewer parameters and MACs, highlighting the balance between complexity and performance. The efficacy of our framework is due to its unique architecture and training process, which includes automatic feature extraction using CNNs and faster training of the BLS, rendering it a competitive solution for X-ray baggage threat segmentation that is adaptable across different datasets and scenarios.

**Table 8:** Model complexity and run-time performance comparison of different methods

Method	Inference time (s)	Parameter (M)	MAC (G)
MS R-CNN [69]	0.156	44.5	141.1
RetinaNet [75]	0.033	34.0	151.5
CIE-Net	0.072	33.4	18.3
YOLOv3 [76]	0.023	63.1	78.6
Mask R-CNN [70]	0.141	44.4	134.3
YOLACT [72]	0.036	34.7	59.2
HTC [71]	0.311	83.2	120.1
**Proposed**	**0.021**	30.8	4.5

### Qualitative evaluations

**Fig. 6:**
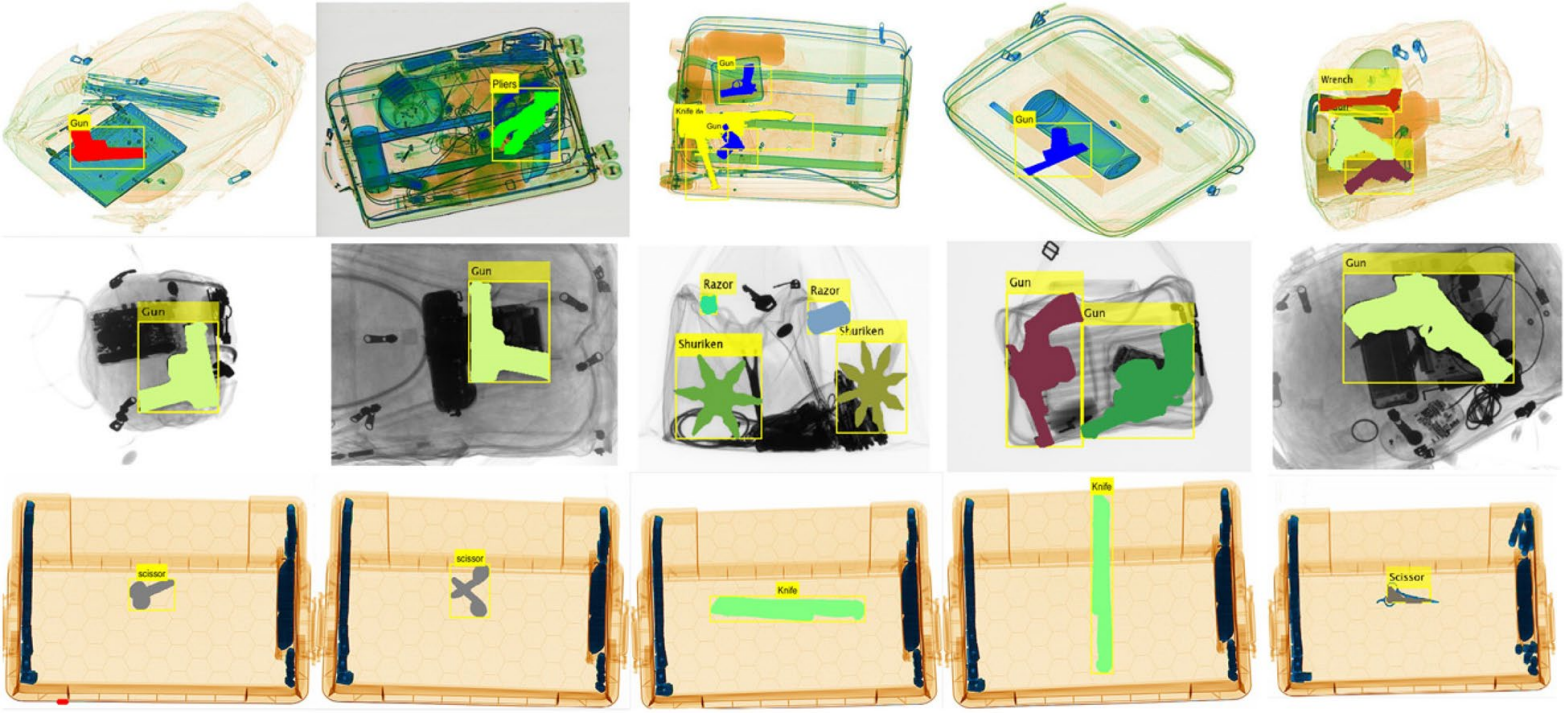
Qualitative evaluations of the proposed framework on SIXray, GDXray and COMPASS-XP

Figure 6 shows the qualitative evaluations of our model on the SIXray, GDXray, and COMPASS-XP datasets. Each row corresponded to a different dataset. The first row highlights the SIXray dataset, where our method successfully identifies single and multiple items, even when hidden. The second row depicts GDXray results, showing effective object identification regardless of the level of exposure. The third row focuses on COMPASS-XP, which demonstrates the capability of the model for single-object detection.

### Failure cases

**Fig. 7:**
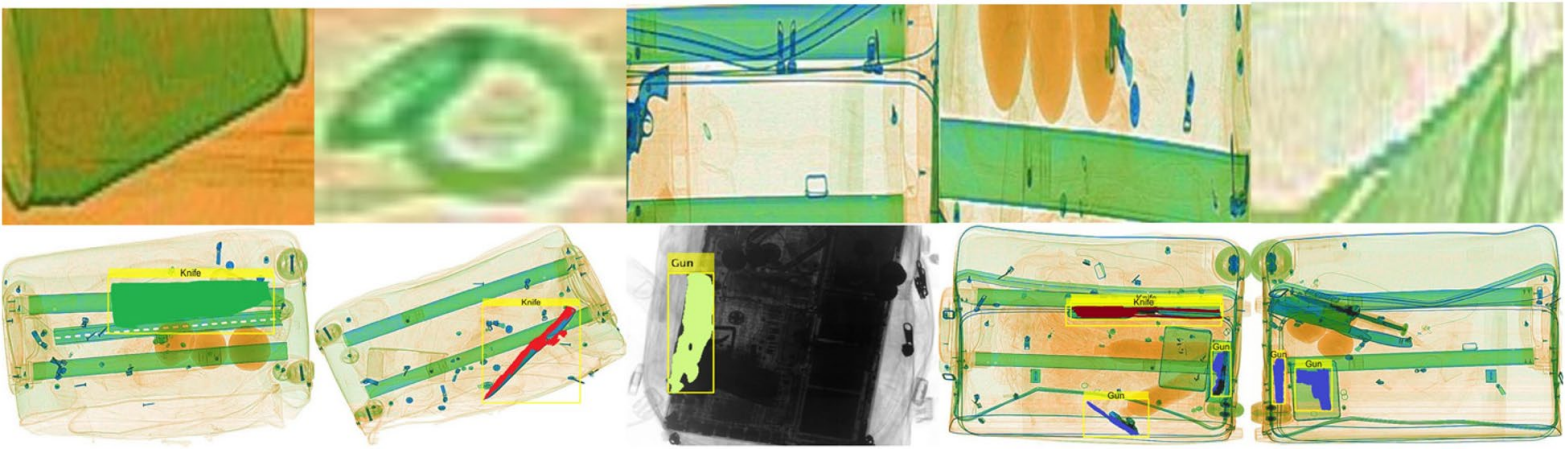
Illustration of failure cases

Figure 7 shows several failure cases from our experiments. The first row illustrates issues with region segment extraction, where mixed segments (with parts of both normal and suspicious items) lead to false positives. The second row displays scenarios in which detection and segmentation are inadequate. Examples include masks containing positive and negative items, incomplete object masking, and undetected objects. Although these failures are rare, they highlight areas of improvement. Post-processing techniques, such as blob filtering, region opening, and region filling, could potentially address these issues. Despite these limitations, our model remains resilient and effective for handling diverse and complex X-ray scans.

### Statistical significance

To assess the statistical significance of the proposed C-BLX framework, *t*-tests were conducted to compare it with CIE-Net, which was the second-best method identified in our study. These tests follow the methodology outlined by Qureshi et al. [77]. For the analysis, the same dataset of test X-ray images was utilized, comprising a 20% subset of the original SIXray collection, amounting to 211,846 images. All images in this dataset were acquired using three different X-ray scanners to ensure that the samples were independent and consistent in type. The results of the *t*-tests revealed a *P* value of approximately 0.0049, which was significantly below the threshold of 0.01. This indicates that our C-BLX framework significantly outperforms CIE-Net with a 99% confidence level. This low *P* value provides robust evidence of the statistical significance of the performance differential between the C-BLX framework and CIE-Net. This result strengthens our confidence in the C-BLX system’s effectiveness and underscores its superior capabilities in analyzing X-ray imagery for baggage examination in comparison to other leading methods.

## Conclusions

This study explored BLS leveraging deep, low-rank representations to recognize and segment prohibited items from cluttered X-ray baggage scans, regardless of scanner characteristics. Notably, the proposed C-BLX was trained once with limited scans from three datasets, mitigating the need for extensive training data, while achieving comparable performance to other frameworks that were fine-tuned separately. C-BLX demonstrates robustness against bias, handles different scanner specifications, and effectively addresses class imbalance. Training with minimal supervision using resource-efficient image-level labels avoids the labor-intensive mask-level annotations required by competing models. The RCR block generates several candidate segments from a single scan, thereby ensuring sufficiently balanced training data. C-BLX achieved impressive results on three public datasets, with mIoU scores of 90.04%, 78.92%, and 59.44% on GDXray, SIXray, and COMPASS-XP, respectively, surpassing those of competing frameworks. In addition, it excels in runtime performance, balancing accuracy, and efficiency, making it a practical solution for aviation security.

Despite these promising results, several enhancements should be explored in future studies. C-BLX is not explicitly designed for continual learning. Therefore, developing models that support lifelong learning is crucial for adapting to emerging threats. In addition, incorporating graph neural networks within the RCR block can improve the identification and refinement of candidate regions. Exploring vision transformers as a replacement for CNNs may further enhance their performance owing to their effectiveness in various visual tasks. Finally, investigating the resilience of C-BLX to noisy inputs could improve the robustness of real-world scanning environments.

## Data Availability

The datasets used in this work are publicly available. They can be accessed through the following links: SIXray: https://github.com/MeioJane/SIXray; GDXray: http://dmery.ing.puc.cl/index.php/material/gdxray/; COMPASS-XP: https://zenodo.org/records/2654887#.YUtGVHVKikA.

## Abbreviations

CT: Computed tomography
BLS: Broad learning system
RCR: Recursive candidate refinement
SOTA: State-of-the-art
*µ*DC: Mean dice coefficient
*µ*IoU: Mean intersection-over-union
MC-BLS: Multi-convolutional broad learning system
PCA: Principal component analysis
CNN: Convolutional neural network
RPN: Region proposal network
MAC: Multiply-accumulate operation
AP: Average precision
µAP: Mean average precision
DC: Dice coefficient
